# Sexual function in Chinese women from pregnancy to postpartum: a multicenter longitudinal prospective study

**DOI:** 10.1186/s12884-021-03546-6

**Published:** 2021-01-19

**Authors:** Qiuxiang Zhang, Min Shen, Yaning Zheng, Shimei Jiao, Shangxiao Gao, Xiaoling Wang, Li Zou, Miao Shen

**Affiliations:** 1 Department of Nursing, Nanjing Drum Tower Hospital, The Affiliated Hospital of Nanjing University, Nanjing, 210008 Jiangsu China; 2 Department of Nursing, The Affiliated Hospital of Yangzhou University, Yangzhou, China; 3 Department of Obstetrics and Gynecology, Huaian Maternal and Child Health Hospital, Huaian, China; 4 Nanjing Drum Tower District Maternal and Child Health Center, Nanjing, China

**Keywords:** Sexual function, Chinese women, Prospective study

## Abstract

**Background:**

The aims of our research were as follows: First, to estimate the prevalence of female sexual dysfunction in early, middle, late stages of pregnancy, and postpartum 6 months after delivery. Second, to discuss relevant factors associated with female sexual dysfunction among women in 6 months after delivery in Nanjing, Yangzhou and Huaian Main, China.

**Methods:**

Our multicenter longitudinal study was carried out from September 2017 to March 2019, with participants recruited from Southeast China: Nanjing, Yangzhou and Huaian. Participants were recruited when they built their Record of Prenatal Care in community hospitals. The online questionnaires included a set of validated tools, sociodemographic information as wells as medical history data. In the meantime, qualitative interviews were conducted during different periods of pregnancy (from the first trimester to the third trimester of pregnancy and following up to six-month postpartum) respectively. All participants have obtained written informed consent.

**Results:**

By qualitative interview, the vast majority of the participants were inactive in having sex from pregnancy to postpartum. There were negative aspects of sexual experiences, emotional responses closely related to self-attitudes toward sexual behavior during this period. Through quantitative analysis, pre pregnancy BMI (OR = 1.15, *P* = 0.012), postpartum weight gain (OR = 1.057, *P* = 0.033) and partnership quality (OR = 1.181, *P* = 0.04) were associated with postpartum sexual dysfunction 6 months after delivery.

**Conclusions:**

Women are at the risk of significantly different FSD with regard to pre-pregnancy BMI, postpartum weight gain and partnership quality. The impaired sexual function from pregnancy to postpartum period indicated the requirement for further survey as well as extensive investigation.

**Supplementary Information:**

The online version contains supplementary material available at 10.1186/s12884-021-03546-6.

## Background

Sexual function has played a very important role in women’s quality of life, which is defined by the World Health Organization as “a state of physical, mental, emotional and social well-being related to sexuality” [1]. Sexuality is a natural part of human life and a multidimensional concept affected collaboratively by hormonal milieu, psychological, social, interpersonal relationships as well as cultural elements. On the whole, sexual function relatively descends when they are pregnant, which also continuously keeps a low level during the postpartum period for many women. Systematic reviews and qualitative studies have demonstrated that decrease in frequency of desire, satisfaction and sexual intercourse occurs during the third trimester compared with that during the pre-pregnancy period. Furthermore, the duration of intercourse and ability to experience orgasm decline in the later stage of pregnancy compared with that in pre-pregnancy. Meanwhile, the difficulty in having sex during pregnancy increases significantly [2–4].

Literally speaking, there was a decrease in coital frequency from the first to third trimesters during pregnancy. A considerable number of physical and psychological elements may result in the reduction in having sex. As far as physical changes were concerned, hormonal variation could raise progesterone level, prolactin and estrogen, which were considered to be responsible for symptoms of vomiting, nausea, and breast tenderness. Meanwhile, anxiety, fatigue and dissatisfying body image resulted from weight gain could bring about general malaise and difficulty in self-awareness [2, 5]. Previous researches have demonstrated such emotional responses as “fear of inducing a miscarriage”, “fear of preterm labor”, “fear of the onset of labor”, “fear of bleeding”, and “fear of an infection” [6–9].

Although there is a lack of large prospective researches, it is generally acknowledged that women frequently have difficulty in sex during the postpartum period. Existing researches have shown a prevalence ranging from 30 to 60% in the first 3 months of postpartum period, falling to 17–31% 6 months after giving birth to a baby [2, 10, 11]. Certain studies have revealed that both mode of delivery, hormonal state, breastfeeding status, and psychosocial variables (fatigue, depression, stress, body image, sleep deprivation, social support, partnership quality, and etc) may contribute to postpartum dysfunction [12–14].

Longitudinal studies regarding maternal dysfunction with long-term follow-up are scarce, relevant information concerning Chinese women in particular. Researches in nonpregnant women indicated that there was a significant distinction in attitude towards having sex between westerners and Chinese [15]. Regardless of various living environment, Chinese women are relatively more conservative about premarital sex and holding a conventional attitude towards having sex. The limited data in Chinese women regarding this part provides medical workers with little reference. Consequently, we conducted a multicentral longitudinal prospective research to investigate sexuality of Chinese women. The purpose of our research is multifaceted. First, to estimate the prevalence of female sexual dysfunction in early, middle, late stages of pregnancy, and postpartum 6 months after delivery. Second, to discuss relevant factors associated with female sexual dysfunction among women in 6 months after delivery in Nanjing, Yangzhou and Huaian Main, China.

## Methods

### Participants

Our multicenter study was conducted in Gulou District, Nanjing, in the maternal and child health center, department of obstetrics and gynecology in Nanjing drum tower hospital, department of obstetrics and gynecology in affiliated hospital of Yangzhou University, and Huaian maternal and child health hospital, from September 2017 to March 2019. This study was permitted by the research ethics committee of hospitals involved and the approval number was 2018-20ZSC530. We designed a four-time-point follow-up prospective study to investigate maternal sexuality. Our participants were included when they established Prenatal Examination Record Card in the community hospital. Subsequently, those pregnant women came to the corresponding comprehensive hospital for delivery. Finally, we conducted follow-up surveys for eligible participants who met the criteria.

Eligibility criteria were: (1) 18 years of age or older, singleton pregnancy; (2) delivery of babies in good physical condition (3) having normal cognition and participating in the research based on her own will. Exclusion criteria were: (1) having cognitive or hearing impairment; (2) having chronic diseases or other complications (hypertension, diabetes, heart disease, bleeding and preterm labor etc) in pregnancy that may seriously influence sexual function and general quality of life; (3) without a partner or non-heterosexual; (4) not completing the questionnaires thoroughly. During the study period, there were about 1000 eligible women in the participating hospitals and about 600 women were approached for the recruitment. Only 279 women volunteered to participate in the study, 33 of them can’t finish assigned tasks at certain time nodes, 29 of the participants were excluded due to postpartum complications. Eventually 217 individuals participated in our study. When women established their prenatal examination record card in the community hospitals, the research procedure was explained. After completing the questionnaires, we interviewed some of the women according to the feedback from questionnaires. During different periods of pregnancy, we conducted qualitative interviews and questionnaires respectively. A few months later, those pregnant women came to the corresponding comprehensive hospital for delivery. The participants agreed to fill in the questionnaires online.

## Main outcome measurements

The online questionnaires including a set of validated tools, socio-demographic data as wells as medical information were employed. The validated Female Sexual Function Index (FSFI), consisting of 19 questions, was used to measure women’s sexual function [16]. The FSFI questionnaire covers six sexual dimensions: lubrication, desire, orgasm, arousal, pain and satisfaction. The FSFI questionnaire was marked with credits ranging from 2 to 36, with higher scores related significantly to better sex function. Participants who scored less than 26.55 were considered to experience female sexual dysfunction (FSD) [17]; The Edinburgh Postnatal Depressive Scale (EPDS) was made to survey depression symptom, which has been confirmed to be sensitive and specific in predicting depression [18]. Answers of scale were based upon psychological status over the past 7 days; Fatigue degree was evaluated on the basis of the multidimensional fatigue inventory-20 (MFI-20) [19]; The Social Support Rating Scale (SSRS) to estimate social support level is demonstrated, which has been used and verified among different Chinese populations [20]; Body image was investigated through Body Image Self-Consciousness Scale; The visual analogue scale was used to estimate women’s global assessment of partnership quality, with the degree of ranging from 0 to 10: 0 = not satisfied at all, 5 = moderate satisfaction, and 10 = complete satisfaction. Those scales (such as FSFI [21], EPDS [22], MFI-20 [23], SSRS [24], Body-image self-consciousness scale [25], and visual analogue scale [26]) have been validated in mainland China with simplified Chinese language. Meanwhile, data concerning delivery, breastfeeding and other relevant information were acquired from medical records as well as follow-up questionnaires after delivery.

In the meantime, qualitative interviews were conducted during different periods of pregnancy respectively (Table 1). The purpose of this section was to raise open-ended questions so that respondents can elaborate on their personal experiences. Information was collected from semi-structured and in-depth interviews conducted among 39 healthy pregnant women. This qualitative interview came to an end when three consecutive participants were unable to provide any new themes, which demonstrated this qualitative interview had reached a saturation point.

**Table 1:** Content of interview outline

Item	Content
Pregnancy related	1. Could you tell me when did you get pregnant?
	2. Was this pregnancy within the scope of the plan?
	3. Do you have any personal thoughts on the current pregnancy?
The impact of pregnancy on life and family	4. How did the pregnancy affect your state of life?
	5. How did the pregnancy affect your family or loved ones?
	6. What changes did your body have during pregnancy?
	7. What are your psychological or emotional changes during pregnancy?
Effects on sexual life and sexual function	8. How did you feel from early pregnancy to postpartum? Could you talk?
	9. Could you talk about some sexual life or sexual function changes during your pregnancy?
	10. Could you talk about some sexual life or sexual function changes in the postpartum period? Can you talk?
	11. What are the changes in your sex life during the first trimester to postpartum respectively?
	12. How did your husband perform from pregnancy to postpartum? Are you satisfied with his performance?

### Statistical analyses

The investigation data were fed into the computer database by two independent assistants who studied the anonymous data after rechecking them. SPSS version 21.0 was operated to perform the statistical analyses for our research. Descriptive analysis was presented with mean (± standard deviation) or number (percentage) according to parametric distribution of variables. Demographic and psychological variables were screened through univariate tests of the group difference (FSD versus non-FSD based upon FSFI≤26.55) at a lenient level of significance (alpha value = 0.05).Chi-square test was applied in analyzing differences in proportion. Chi-square test was applied in analyzing differences in proportion.to assess mean differences, and we also use to investigate how variables might collectively influence female sexual function. Variables significantly related to sexual function were included in the logistic regression model by univariate testing. FSFI was evaluated as a dependent variable with the dichotomous female sexual function.

## Results

### Participant characteristics

Eventually, a total of 217 participants were involved in the research with an average age of 29.11 years old (SD = 4.21). Demographic and clinical variables were as the follows: age, education level, occupation, monthly income, pre-pregnancy BMI, postpartum weight gain, reproductive history, mode of delivery, and breastfeeding characteristics. Further assessed demographics can be seen in Table 2.

**Table 2:** Participant Characteristics

Participants’ data (*n* = 217)	Value
Maternal age, mean ± SD	29.11 ± 4.21
Education level, n (%)	
Primary (0–6 yrs)	20(9.22%)
Secondary (7–12 yrs)	27(12.44%)
Higher (> 12 yrs)	170(78.34%)
Employment	
Employed	176(81.11%)
Housewife/unemployed	41(18.89%)
Monthly income, n (%)	
< 3000	33(15.21%)
3000–8000	90(41.47%)
> 8000	94(43.32%)
Pre pregnancy BMI (kg/m2)	21.16 ± 2.84
Postpartum weight gain (kg)	9.52 ± 6.21
Reproductive history	
Primipara	144(66.36%)
Multipara	73(33.64%)
Mode of delivery, n (%)	
Vaginal	148(68.20%)
Caesarean	69(31.80%)
Breastfeeding characteristics	
Exclusively	86(39.63%)
Mixed	98(45.16%)
Artificial	33(15.21%)

### Qualitative interview analysis

According to the interview outline (Table 1) of qualitative research, we found that no increase in coital frequency during pregnancy period and also a sharp decline in frequency. The vast majority of pregnant Chinese women tended to stop having vaginal intercourse from the first to third trimester respectively. Thinking of it as relatively beneficial to them for not having vaginal intercourse while she was pregnant. Sex was strictly avoided from the first to the third trimester in particular. Negative influences of having sex on women were as follows: (a) feeling too heavy to move body; (b) pain of pelvis; (c) fatigue or tiredness; (d) discomfort during coitus (e.g. uterine contractions and, dry vaginal mucosa).

The subjects’ emotional responses were closely associated with self-perceptions and attitudes toward sexual activities: 1) fear, including (a) the thought that coitus was dangerous which could hurt the fetus; (b) the worry about bleeding, miscarriage, early birth, and vaginal infection. 2) anxiety, including (c) gain in weight, being out of shape and reduction in attraction during pregnancy; (d) partner’s incomprehension, which might lead to dissatisfaction with sexual life; (e) instability of marriage. Influenced by social, cultural, and religious factors of China, they seldom took the initiative to discuss about it. The participants tended to get information regarding sexual activity from the Internet and the postpartum women around them. Few of them discussed with their doctors concerning this topic before, nor did they mention the subject actively.

### Prevalence of sexual dysfunction and descriptive statistics during different period

Table 3 analyzed the last 217 women with complete data. Table 3 summarized sexual function of women enrolled during each period. Two hundred eleven women (97%) elaborated an overall decline of sexual activity during pregnancy, whereas four (2%) reported almost unchanged, and only two (1%) that there had been an increase in their sexual activity while pregnant (Not shown in the table). The vast majority who reported sexual activity in the first 3 months of pregnancy continued to be surveyed in the second and third trimester. Some pregnant women were still undergoing vaginal intercourse. Compared with that in the first or second trimester of pregnancy, the sexual activity in the third trimester decreased significantly. After analyzing the data, we found that based on the cut-off point of FSFI = 26.55, the prevalence of FSD in 1st trimester was 100% (*n* = 217), 2nd trimester was 97.23% (*n* = 211), 3rd trimester was 96.21% (*n* = 203), postpartum 6 M was 64.06% (*n* = 139) in Table 3.

**Table 3:** Prevalence of sexual dysfunction and descriptive statistics during different period

Assessment (*n* = 217)	Desire		Arousal		lubrication		Orgasm	Satisfaction			Pain	Total score			FSD	
	M	SD	M	SD	M	SD	M	SD	M	SD	M	SD	M	SD	n	%
1st trimester	2.84	0.89	2.67	1.15	3.50	1.14	2.77	1.09	2.88	1.09	3.06	1.13	17.72	2.63	217	100%
2nd trimester	2.96	1.08	3.56	1.30	3.44	1.22	3.08	1.04	3.55	1.25	3.12	1.08	19.73	3.21	211	97.23%
3rd trimester	2.35	1.12	1.63	1.67	1.51	2.07	1.33	1.92	2.84	1.77	1.50	2.20	11.17	8.62	203	96.21%
Postpartum 6 M	4.24	1.01	4.60	0.98	4.52	0.99	3.76	1.05	4.42	0.87	3.77	1.13	25.31	2.51	139	64.06%

Table 3 analyzed the last 217 women with complete data

### Differences between non-FSD and FSD women

The social-demographic, psychological and clinical differences between non-FSD and FSD women 6 months after delivery were presented in Table 4. Education level, employment condition, pre-pregnancy BMI, postpartum weight gain, and partnership quality were found to be associated with postpartum sexual dysfunction 6 months after delivery(*P* < 0.05). With regard to age, monthly income, reproductive history, mode of delivery, breastfeeding characteristics, postnatal depression, social support, body image, sleep quality, pain, and fatigue, no significant statistical differences were found between these two groups (*P* > 0.05). Although the effect on body image (*P* = 0.096) and pain (*P* = 0.09) was as what had been expected, there were no significant statistical differences on the basis of current data.

**Table 4:** Differences between non-FSD and FSD women (during six-month after delivery)

Characteristics	Non-FSD1(*n* = 87)	FSD(*n* = 130)	*P*-value
Maternal age, mean ± SD	28.89(4.02)	29.26(4.35)	0.520
Education level, n (%)			0.023
Primary (0–6 yrs)	6(6.89%)	14(10.77%)	
Secondary (7–12 yrs)	5(5.75%)	22(16.92%)	
Higher (> 12 yrs)	76(87.36%)	94(72.31%)	
Employment			0.054
Employed	11(12.64%)	30(23.08%)	
Housewife/unemployed	76(87.36%)	100(76.92%)	
Monthly income, n (%)			0.690
< 3000	11(12.64%)	22(16.92%)	
3000–8000	37(42.53%)	53(40.77%)	
> 8000	39(44.83%)	55(42.31%)	
Pre pregnancy BMI (kg/m2)	20.58(2.47)	21.54(3.01)	0.011
Postpartum weight gain (kg)	8.19(5.35)	10.41(6.69)	0.007
Reproductive history			
Primipara	62(71.26%)	82(63.08%)	0.211
Multipara	25(28.74%)	48(36.92%)	
Mode of delivery, n (%)			0.920
Vaginal	59(67.82%)	89(68.46%)	
Caesarean	28(32.18%)	41(31.54%)	
Breastfeeding characteristics			0.815
Exclusively	36(41.38%)	50(38.46%)	
Mixed	37(42.53%)	61(46.92%)	
Artificial	14(16.09%)	19(14.62%)	
Hospitals, n (%)			0.834
Nanjing	37(42.53%)	51(39.23%)	
Yangzhou	32(36.78%)	53(40.77%)	
Huaian	18(20.69%)	26(20.00%)	
Postnatal Depression, mean ± SD	5.94(8.96)	4.91(5.01)	0.335
Social support, mean ± SD	38.19(9.45)	37.22(8.03)	0.429
Body image, mean ± SD	15.54(6.93)	17.29(8.44)	0.096
Partnership quality, mean ± SD	8.19(1.99)	7.51(1.93)	0.014
Sleep quality, mean ± SD	7.29(2.95)	8.04(3.85)	0.106
Pain, mean ± SD	6.73(2.13)	6.22(2.18)	0.090
Fatigue, mean ± SD	48.52(12.19)	51.08(11.75)	0.121

*FSD* female sexual dysfunction

### Logistic regression analysis of postpartum sexual dysfunction

Logistic regression analysis was used to explore predictors of postpartum sexual dysfunction 6 months after delivery. As indicated in Table 5, we drew the conclusion that pre pregnancy BMI (odds ratio = 1.15; *P* = 0.012), postpartum weight gain (odds ratio = 1.057; *P* = 0.033), and partnership quality (odds ratio = 1.181; *P* = 0.04) were the predictors of postpartum sexual dysfunction.

**Table 5:** Logistic regression analysis of postpartum sexual dysfunction (six-month after delivery)

Variables	B	S.E,	Wals	Sig.	Exp (B)	95% CI	
						Lower	Upper
Education level	−0.46	0.263	3.054	0.081	0.632	0.378	1.057
Employment	−0.51	0.409	1.568	0.211	0.600	0.269	1.335
Pre pregnancy BMI	0.14	0.055	6.363	0.012	1.150	1.032	1.281
Postpartum weight gain	0.06	0.026	4.531	0.033	1.057	1.004	1.113
Partnership quality	0.17	0.080	4.229	0.040	1.181	1.008	1.381
Body image	0.02	0.020	1.431	0.232	1.024	0.985	1.065
Pain	−0.01	0.007	0.852	0.356	0.993	0.979	1.007

## Discussion

Sexuality is a natural part of human life as well as a multidimensional concept jointly influenced by hormonal milieu, psychological, social, interpersonal relationships and cultural elements [12]. A prospective cross-sectional study of pregnant women conducted in Hong Kong found that vaginal intercourse significantly decreased during the third trimester. In addition to gestation, advanced maternal age and nulliparity were also independent factors related to reduction in vaginal intercourse [9].

In terms of postpartum period, different studies reported inconsistent results. Hipp L. E. et al. [27] have reported that women’s postpartum sexual desire was affected by their perceptions of partner’s postpartum sexuality and individual’s degree of fatigue. Postpartum desire was not significantly affected by vaginal issues, breastfeeding status, or social psychological condition including stress, social support or body image. Shirvani M. A. et al. [28] have found that sexual function had significant association with longer marriage duration, older maternal age, and larger number of children. Mothers disease, neonate problems and tuboligation were related to lower scores of sexual activities. There was no correlation between perineal injuries or mode of delivery. Chang S. R. et al. [13] have investigated that the cesarean birth group had a significant higher prevalence of depression, higher scores of pain, lower sexual satisfaction scores. Jawed-Wessel S. et al. [29] showed that there was significant correlation between the body satisfaction, body image self-consciousness, and female sexual function. According to Faisal-Cury A. et al., [30] such variables as anxiety/ depressive symptoms during both pregnancy and postpartum, previous miscarriage and age of pregnant were independently related to decline in having sex. Wallwiener S.et al. [31] suggested that women who were at the risk of experiencing sexual dysfunction differed significantly in terms of mode of delivery, breastfeeding status, partnership quality, maternal education, and depression condition. A prospective cohort study made by Lagaert L. et al. [14] in Belgium, in the first 6 weeks of postpartum, degree of dyspareunia was significantly related to breastfeeding status and primiparity. Six months after delivery, only the primiparity played a leading role in having sex. A longitudinal prospective study conducted regarding female sexual function prior to pregnancy, at enrollment, and at 2, 6, 12, and 24 weeks postpartum in United States of America, which showed that episiotomy or mode of delivery were not associated with intercourse resumption, while dyspareunia was only related to breastfeeding at 12 weeks [11].

The present study investigated sexual function over a period from the first trimester to the third trimester of pregnancy and following up to six-month postpartum participants recruited from Southeast China. Through qualitative interview, we have found the vast majority of women were either sexually inactive or showed FSFI scores indicative for FSD while being assessed at any point from pregnancy to postpartum. There were negative aspects of emotional responses, sexual experiences closely associated with self-perceptions and attitudes toward sexual behavior during pregnancy. Few of them discussed with their doctors concerning this topic before, nor did they mention the subject actively. Interestingly, we also conducted quantitative analyses and found that education level, employment, pre pregnancy BMI, postpartum weight gain and partnership quality were closely associated with postpartum sexual dysfunction 6 months after delivery. With regard to age, monthly income, reproductive history, mode of delivery, breastfeeding characteristics, postnatal depression, social support, body image, sleep quality, pain, and fatigue, no significant statistical differences were found between two groups involved. Although the effect on body image and pain were in the expected direction, there were no statistical significance based upon current information. In addition, we found that pre-pregnancy BMI, postpartum weight gain and partnership quality were the predictors of postpartum sexual dysfunction. In our traditional culture, sex culture is conservative and implicit. Women’s attitudes towards marriage and sex are conservative and restrained. The intimate relationship and understanding of heart are the best lubricants between husband and wife. Consequently, the partnership quality counts. A cross-sectional research involving 223 pregnant women conducted by Ribeiro M C et al. [32] in Brazil demonstrated that there was a negative correlation among pre-pregnancy BMI, mean 3rd trimester, total FSFI scores and orgasm. Overweight women in the 3rd trimester of pregnancy had poorer sexual function compared with pregnant women with normal weight [32]. Under the cultural background of slimness as beauty and the second child policy, more and more women pursue slimness. The surge in weight during pregnancy and post-natal body distortion have added heavy blow to women, which also reduce the attraction of wife in husband’s heart to some extent. These results were consistent and inconsistent with the studies mentioned above. This might be ascribed to genetic and environmental backgrounds differences with relevant cultural factors. Possibly, women groups with different inclusion and exclusion criteria may account for our results. On the other hand, we found that FSD prevalence was surprisingly high during pregnancy (100% prevalence during the first trimester, 97.23% the second trimester, 96.21% the third trimester). There was a slight improvement in the postpartum stage (FSD = 64.06%). This may be related to the conservative traditional concept of Chinese women. In their concept of fertility, fetal health was the most important, which maintained the hope of whole family. The first and third trimester were the most dangerous periods, and there should be no relaxation. But in the postpartum, because of the baby’s safe birth and physical recovery, they gradually begin to pay attention to themselves.

As we have seen, our research may be the first relatively comprehensive concerning the potential risk factors of female sexual function in Southeast China during different periods that have been conducted. Meanwhile, our study also had several limitations: First, although participants involved in this research came from multiple centers, the number of samples was relatively small and had no control group. Second, the measurements being employed to make assessment were subjective to a certain extent, which may lead to possible deviation. Third, the FSFI was for women who have been sexually active in the last 4 weeks, some of us population were not suitable. Although we conducted qualitative interview to further elaborate, to some extent, there was still shortcoming. Meanwhile, due to the influence of time and funds, we only studied 6 months after delivery, and failed to conduct longer follow-up.

## Conclusion

Findings suggested that women at risk of FSD significantly differed in terms of pre-pregnancy BMI, postpartum weight gain and partnership quality. The impaired sexual function during the period from pregnancy to postpartum indicated the requirement for further survey as well as extensive consultation.

## Supplementary Information

**Additional file 1.**

## Data Availability

Our raw data could be found in the supplementary files.

## Abbreviations

FSD: Female sexual dysfunction
BMI: Body Mass Index
FSFI: Female Sexual Function Index
EPDS: Edinburgh Postnatal Depressive Scale
MFI-20: Multidimensional fatigue inventory-20
SSRS: The Social Support Rating Scale
